# A Regrettable Misunderstanding

**Published:** 1920-03-13

**Authors:** 


### THE TREATMENT OF THE INSANE SOLDIER.

### A Regrettable Misunderstanding.

In the matter of the treatment of the insane soldier the authorities were sadly at sea in the early part of the war. Officially it was realised that soldiers did become insane, and there were regulations dealing with them. Later on there was a rapid increase in the number of soldiers mentally affected, and the authorities were unprepared for dealing satisfactorily with them. The increase was much greater than it need have been if some provision for the proper examination of recruits from the mental point of view had been arranged for. Many of these mentally affected were said by their friends to be suffering from "shell-shock," a blessed euphemism comparable to "nervous breakdown," or "neurasthenia" as popularly understood. A demand arose that these patients were not to be treated as insane, they were not to be certified, nor were they to be put in charge of those who had experience of the care and treatment of the insane. Special hospitals accordingly were arranged for the reception of these cases.

So far, so good; but the sentimentalists did not realise that, whatever name might be given to the disorder from which many of these patients suffered, the fact remained that they were insane. Many had already spent periods in asylums, and others were of that unhappy band who, war or no war, were in any case doomed to swell the asylum population. In due course these patients were drafted on to the asylums—that is to say, to places specially equipped for the proper supervision and care of the insane. This—and the alleged ill-treatment in military hospitals—is now exercising the minds of certain people, in particular the writer of articles in *Truth* on "Army Mental Hospitals," etc. (January 28 and February 4, 11, and 18). He draws a lurid picture of the sad lot of patients in some Army hospitals, and speaks of the "dark cells and padded room of the lunatic asylum, with brutal warders cowing the unfortunate occupants by physical force," and so on; and he asks us to compare this with the routine in another place where "patients had full liberty to come and go, means of amusement were liberally provided, their friends had access to them, the nursing was in the hands of skilled women nurses."

It is an antithesis quite likely to appeal to the uninstructed; but the description is inaccurate, and in the former instance is a needless slur upon a body of men who have fulfilled their duties sympathetically and patiently under trying circumstances. No one would be foolish enough to deny that instances of ill-treatment by individual attendants and orderlies may have occurred. There are black sheep in every flock; and some men—and, indeed, some women—are unsuitable for the duty of attending upon the insane. When this is found to be so they are dealt with by those in authority, drastically if occasion requires. But to say that the few complaints—and they are few in comparison with the large numbers of patients passing through the various hospitals—typify the conduct generally in these hospitals and in asylums is absurd and dishonest. Then, too, all kinds of mental disorder have had to be treated. The cases have *not* been indiscriminately herded together.

It seems to be forgotten that the Army has gathered in not only many of the finest types of humanity, but also some of the dregs of the population. All these were represented among the insane. Many of the patients could soon be granted all kinds of privileges: others were quite unfit. Among the latter were epileptics, men suffering from chronic alcoholism of the delusional type and from general paralysis (in the excited stage), paranoia, and so on. Some of these were violent and even murderous. No sane person who understood insanity would take risks with such men, at any rate during the bad phases. And these were the cases which, in many instances, had to be moved from the avowedly "shell-shock" hospitals to other places where they could be efficiently safeguarded.

Much more might be said on other points raised by the writer of the articles in *Truth* and by others who have assailed the War Office, and, of course, the Board of Control. This must, in the meantime, suffice. It is a pity, however, that a record of the work done for the soldiers suffering from nervous and mental disorders could not be given to the public. It would then be seen that, taken altogether, a very efficient system for the care of these patients steadily evolved.

